# Associations between appetite, physical activity and sedentary behaviour from hip‐ and wrist‐worn accelerometers in community‐dwelling older adults

**DOI:** 10.1111/ggi.14588

**Published:** 2023-04-26

**Authors:** Li‐Tang Tsai, Eleanor Boyle, Sussi F Buhl, Gry Kock, Jan C Brønd, Marjolein Visser, Nuno Mendonça, Eric J Shiroma, Paolo Caserotti

**Affiliations:** ^1^ Research Unit for ORL—Head and Neck Surgery and Audiology Odense University Hospital Odense Denmark; ^2^ Department of Clinical Research University of Southern Denmark Odense Denmark; ^3^ Clinical Biomechanics Unit, Department of Sports Science and Clinical Biomechanics University of Southern Denmark Odense Denmark; ^4^ Research Unit of General Practice, Department of Public Health University of Southern Denmark Odense Denmark; ^5^ Steno Diabetes Centre Odense Odense University Hospital Odense Denmark; ^6^ Centre of Research in Childhood Health, Department of Sports Science and Clinical Biomechanics University of Southern Denmark Odense Denmark; ^7^ Department of Health Sciences, Faculty of Science, Amsterdam Public Health Research Institute Vrije University Amsterdam Amsterdam The Netherlands; ^8^ Department of Internal Medicine, Nutrition and Dietetics VU University Medical Center Amsterdam The Netherlands; ^9^ EpiDoC Unit, CHRC, NOVA Medical School NOVA University Lisbon Lisbon Portugal; ^10^ Laboratory of Epidemiology and Population Sciences National Institute on Aging Baltimore Maryland USA; ^11^ Center for Active and Healthy Ageing, Department of Sports Science and Clinical Biomechanics University of Southern Denmark Odense Denmark

**Keywords:** exercise, nutrition, wearables

## Abstract

**Aim:**

The objective of the current study was to examine whether physical activity and sedentary behavior were associated with appetite among community‐dwelling older adults.

**Methods:**

Cross‐sectional analysis was performed on three cohort studies: the Longitudinal Aging Study Amsterdam (LASA); the Health, Aging and Body Composition Study (HABC Study) and the I'm Still Standing Study (ISS Study); (*n* = 1173, *n* = 162, *n* = 125; age range: 57–99, 85–95, 80–100 years; women: 51%, 56%, 61%, respectively). Physical activity and sedentary behavior were measured using hip‐worn (LASA and HABC) and wrist‐worn (ISS) accelerometers. Appetite was self‐reported. Logistic regression models were fitted by accelerometer placement to explore the association between good appetite and various physical activity metrics (total activity, sedentary behavior, and time spent in different intensities of physical activity).

**Results:**

Among cohorts using hip‐worn accelerometers, those having total activity within the highest tertile had more than double the odds of having good appetite compared with those within the lowest tertile (odds ratio [OR] 2.16 (1.15–4.06)). Each additional percent of daily sedentary behavior decreased the odds for having good appetite by 3% (OR 0.97 (0.95–0.996)), while each additional percent of daily light‐intensity physical activity increased the odds for having good appetite by 4% (OR 1.02 (1.01–1.06)). No association was found between either physical activity or sedentary behavior and appetite for measurements with the wrist‐worn accelerometers.

**Conclusions:**

Among community‐dwelling older adults, the associations between appetite, accelerometer‐assessed physical activity and sedentary behavior differ by accelerometer placement location. This study highlights the importance of careful interpretation of accelerometer data from different body locations and concurrent health outcomes. **Geriatr Gerontol Int 2023; 23: 411–417**.

## Graphical abstract

- Physical activity and sedentary behavior
- Hip‐worn accelerometer
- Appetite
- Wrist‐worn accelerometer

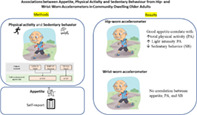

## Introduction

Poor appetite is common among community‐dwelling older adults,^1^, ^2^ with a prevalence of around 30%,^3^ and can lead to decreased food intake and anorexia of ageing, a multifactorial geriatric syndrome.^1^ Poor appetite is strongly associated with weight loss and malnutrition,^4^ increased likelihood of low energy and protein intake,^5^ reduced muscle mass, lower muscle strength^2^ and mobility difficulties.^1^ For older adults, mobility is an indicator for independent living and is strongly associated with physical activity.^6^

Physical inactivity and sedentary behavior are modifiable risk factors for chronic diseases, functional loss and disability.^7^ Traditionally, physical activity and sedentary behavior have been assessed with self‐report methods, which are easy to administer^8^ but prone to recall bias. In contrast, accelerometers are sensitive devices and provide a feasible way to measure physical activity and sedentary behavior objectively in older adults.^9^, ^10^ However, with an increasing number of studies using different accelerometer study protocols,^11^ particularly regarding anatomical wear locations, caution is warranted when comparing studies and different health outcomes.

To date, limited evidence exists on the association between physical activity and appetite among older adults. Physical activity is associated with lowering the risk of developing several physical and mental health conditions, which are also risk factors for appetite loss in older adults, including depression, sarcopenia, and loneliness.^3^ In contrast, physical inactivity can result in lower energy requirements, which may contribute to poorer appetite.^12^ A recent systematic review concluded that there was insufficient evidence to support the association between physical activity and appetite,^13^ highlighting the limited and inconsistent research in this area. Nevertheless, indirect links between appetite and physical activity have been reported, such as association between reduced appetite and low protein intake^5^, which is associaoed with physical inactivity.

To our knowledge, the current study is the first to examine the association between appetite, device‐measured physical activity, and sedentary behavior among older adults. We aimed to examine the association between physical activity, sedentary behavior and appetite as well as whether appetite was independently associated with physical activity and sedentary behavior, as measured with hip‐ and wrist‐worn accelerometers, among community‐dwelling older adults from Europe and the USA.

## Methods

### Study population

This study is part of a larger European research project entitled PROMISS (Prevention Of Malnutrition in Senior Subjects in the EU) and included cross‐sectional analysis of three cohorts: (i) the Longitudinal Ageing Study Amsterdam (LASA, the Netherlands)^14^; (ii) the Health, Aging and Body Composition Study (HABC Study, USA)^15^; and (iii) the I'm Still Standing Study (ISS Study, Denmark).^5^ All studies were approved by local ethical committees, and informed consent was signed by all participants. These studies are described in detail elsewhere.^5^, ^14^, ^15^

Briefly, LASA is a population‐based study aiming to determine predictors and consequences of aging that has been ongoing since 1992/93 (*n* = 3107); it includes men and women aged 55 to 85 years recruited from three geographical areas in the Netherlands.^14^ Two additional cohorts were recruited from the same sampling frames in 2002–2003 (*n* = 1002), and another in 2012–2013 (*n* = 1023). The current study includes data from the main interview of the LASA Wave I, collected between 2015 and 2016. Participants were instructed to wear an accelerometer (ActiGraph GT3X+; ActiGraph inc, Pensacola, FL, USA) for 7 days on their right hip.

The HABC Study is an interdisciplinary study focused on risk factors for functional decline and body composition in healthier older persons aged 70–79 years in 1997–1998 (*n* = 3075, 45% women, 33% African‐American).^15^ The current study used data collected in 2013–2014. Participants wore an accelerometer (ActiGraph GT3X+; ActiGraph inc, Pensacola, FL, USA) for 7 days on their right hip.

The ISS Study aimed to investigate the prevalence of physical frailty in self‐reliant community‐dwelling adults 80 or over in the Municipality of Odense, Denmark (*n* = 147).^5^ In 2017–2018, participants wore an accelerometer (ActiGraph GT3X/GT3X+; ActiGraph inc, Pensacola, FL, USA) for 7 days on their dominant wrist.

## Measures

### Device‐measured physical activity and sedentary behavior

Participants from LASA and HABC wore the accelerometer on the right side of their waist on an elastic belt. Participants were instructed to remove the accelerometer during sleep and water‐based activities. Participants from ISS were instructed to wear the accelerometer on their dominant wrist continuously, except during water‐based activities. Detailed instructions on recording different behaviors in their accelerometer diary (e.g., sleep, awake and nap times) were provided, and participants were encouraged to maintain their usual daily routines during the 7‐day period.

Harmonization of accelerometer data across different brands and models was achieved by processing all raw acceleration data, using the same software packages (ActiLife version 6.4.11 and Propero), into counts per minute, an aggregate metric of total movement per minute. Participants who wore the device for a minimum of 10+ hours on 4+ days were included.^16^ Data were additionally harmonized to exclude sleep and nighttime activity. Details regarding the processing methods are described in the online supplement (eMethods). Total activity (average total daily counts) was calculated and divided into tertiles. Physical activity and sedentary behavior were summed for the accelerometer wear‐time. In addition, times spent in the defined intensities of physical activity and sedentary behavior were calculated using accelerometer wear‐time as the denominator and were expressed in percentages.

In this study, the physical activity intensity categories were defined according to standard, previously validated vector magnitude (square root of the total of the squares of the individual vector components of acceleration, i.e., √(x^2^ + y^2^ + z^2^)) cut‐points specifically for hip‐worn and for wrist‐worn accelerometers.^17^, ^18^ These cut‐points were developed by validation studies against energy expenditure irrespective of body position. There are currently no available cut‐points above sedentary behavior for wrist‐worn accelerometers, and we have pragmatically reported activities at intensities above sedentary behavior by ranges of 2303–4999 and ≥ 5000 cpm.^19^

### Appetite

Appetite was harmonized across the three cohorts from the original four‐ or five‐point scale into the dichotomous “good appetite” and “moderate to poor appetite”.

### Other sociodemographic and health variables

Weight loss and living alone were harmonized across the three cohorts into a dichotomous variable (yes or no). Age and sex were also collected in each study.

Living arrangement was self‐reported in LASA and HABC using the question: “Besides yourself, how many other people live in your household, or do you live alone?” and by the question: “Are you living alone?” in ISS. This variable was then dichotomized into living alone (yes or no). Age and sex were also collected in each study.

Physician‐diagnosed medical conditions were self‐reported from standardized interview questions, and the number of conditions was calculated (from a list of seven conditions in LASA^20^ and HABC,^21^ and a list of 17 conditions in ISS) and transformed into a categorical variable with (i) none; (ii) one; (iii) two or more medical conditions.

Gait speed (m/s) was measured over 3 m in LASA (maximal walking speed), over 20 m in HABC (preferred walking speed) and as part of the short physical performance battery (SPPB)^22^ over 3 m in ISS (preferred walking speed). The presence of slow gait speed (yes or no) was defined as walking <0.8 m/s.^23^

Details regarding harmonization of appetite and other sociodemographic and health variables are described in the online supplement (eMethods).

### Statistical analysis

All statistical analyses were in parallel for hip and wrist. Participant characteristics were described using median and interquartile ranges (IQRs) or percentages by appetite category. Statistical differences by appetite category were tested using a Mann–Whitney U test for continuous characteristics or a chi‐square test for categorical characteristics.

A series of logistic regressions was conducted separately for hip‐worn and wrist‐worn accelerometers to determine if good appetite was associated with device‐measured movement characteristics: total activity (tertiles of average total daily counts) and percentages of time spent in sedentary behavior and higher intensities of physical activity. For each activity metric, three nested models were used to accommodate six covariates in a stepwise fashion. Model I was adjusted for age and sex; model II was additionally adjusted for weight loss and living alone; and model III was additionally adjusted for number of medical conditions and slow gait speed. All models of hip‐worn accelerometers were adjusted for cohort (LASA or HABC). Participants with wrist‐worn accelerometers (ISS) were, on average, older than those using hip‐worn accelerometers (LASA and HABC). To minimize the age effect, we repeated all models among participants ≥80 years old in LASA and HABC to explore whether the association between appetite and movement characteristics differed by accelerometer placement. Assumptions of logistic regression were met. For both hip‐worn and wrist‐worn accelerometers, no interaction was observed between total activity and age or sex. The results of logistic regression analyses are presented as odds ratios (ORs) and 95% confidence intervals (CIs).

Statistical significance was set at *P* <0.05. Analyses were conducted in Propero (custom‐made at the University of Southern Denmark) and Stata 16 (StataCorp 2019. Stata Statistical Software: Release 16. College Station, TX: StataCorp LLC.).^24^

## Results

**Figure 1:**
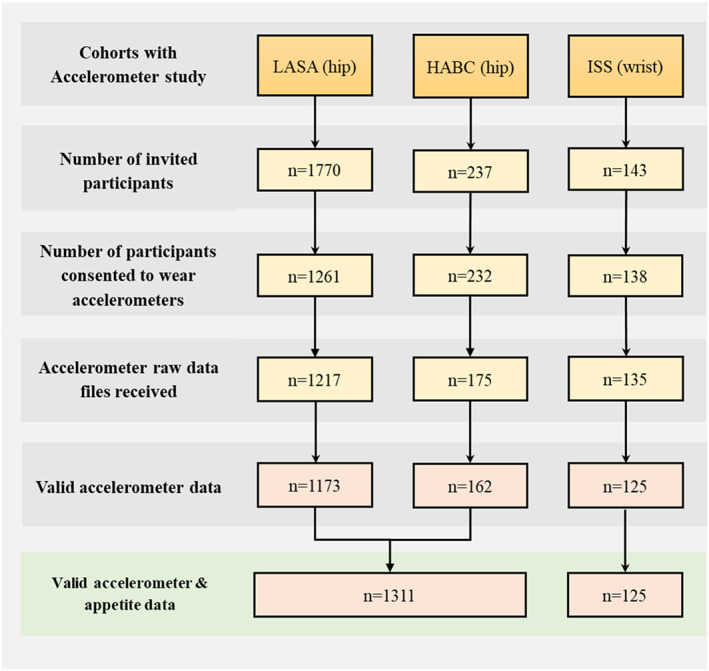
Flow diagram of participants included from the three cohorts. LASA, Longitudinal Aging Study Amsterdam; HABC, Health, Aging, and Body Composition Study; ISS, I'm Still Standing Study.

A total of 2150 participants from LASA, HABC and ISS were invited to participate in accelerometer measurements. Among these, 1631 (76%) accepted the invitation. A total of 1527 accelerometer data files were successfully extracted (from 94% of those who accepted the invitation), among which 1460 had valid accelerometer data (96% of extracted accelerometer data files, 90% of those who accepted the invitation). Finally, participants with valid accelerometer and appetite data were included in the study (*n* = 1311 and 125 with hip‐ and with wrist‐worn accelerometers, respectively) (Fig. 1.).

LASA participants were the youngest with a median age (IQR) of 69 (11) years. HABC and ISS included onlyparticipants ≥ 80 years old (median 87 (4) years for HABC and 86 (4) years for ISS). Prevalenceof good appetite was highest in LASA (92%) and lower in HABC (72%) and ISS (72%).

Participants with hip‐worn accelerometers (median age 71 (14), 51% women, *n* = 1311) had average total daily counts of 492 (395) cpm and wore the accelerometer 14 h 49 min (1 h 30 min)/day. Participants with wrist‐worn accelerometers (median age 86 (4), 61% women, *n* = 125) had average total daily counts of 1836 (716) cpm and wore the accelerometer 14 h 38 min (39 min)/day.

Tables 1a and 1b show participants' characteristics according to appetite strata for cohorts with hip‐worn and wrist‐worn accelerometers, respectively. Prevalence of moderate to poor appetite was 11% among those with hip‐worn and 27% among those with wrist‐worn accelerometers. Among those with hip‐worn accelerometers, the moderate to poor appetite group had 191 fewer cpm than the good appetite group (38% less active, *p* < 0.001). Participants with good appetite accumulated less sedentary behavior (57 (12) vs.61 (16) %, *p* < 0.001) and engaged in more physical activity at light intensity (40 (12) vs. 33 (17)%, *p* < 0.001) and at moderate‐to‐vigorous intensity (2 (4) vs. 0.7 (2) %, *p* < 0.001) compared to those with moderate to poor appetite (Table 1a). In participants with wrist‐worn accelerometers, no significant differences in sedentary behavior and physical activity were observed between those with good appetite and those with moderate to poor appetite (Table 2).

**Table 1:** Participants' characteristics according to appetite strata for cohorts with hip‐worn accelerometers (*n* = 1311)

	Good appetite (*n* = 1170)	Moderate to poor appetite (*n* = 141)	
Cohorts: LASA, HABC	Median (IQR)/%	Median (IQR)/%	*P*‐value^†^
Age (years)	70 (13)	80 (17)	<0.001
58–64	23%	11%	
67–79	55%	36%	
≥80	22%	52%	
Women	50%	65%	<0.001
Presence of weight loss	20%	34%	<0.001
Living alone	24%	39%	<0.001
Chronic conditions			0.115
None	32%	33%	
One	38%	30%	
Two or more	30%	37%	
Slow gait speed	21%	44%	<0.001
Accelerometer wear time (min/day)	14 h 53 min (1 h 27 min)	14 h 20 min (1 h 40 min)	<0.001
Average total daily counts (counts per minute)	505 (280)	314 (277)	<0.001
Total activity			<0.001
High tertile	94%	5%	
Middle tertile	94%	6%	
Low tertile	79%	21%	
Time in SB (0–173) (h, min)	8 h 28 min (2 h 13 min)	9 h 24 min (2 h 40 min)	<0.001
% of time in SB (0–173)	57 (12)	61 (16)	<0.001
Time in light intensity (174–2750 cpm) (h, min)	5 h 55 min (1 h 53 min)	4 h 48 min (2 h 17 min)	<0.001
% of time in light intensity (174–2750)	40 (12)	33 (17)	<0.001
Time in MVPA (≥ 2751) (min)	22 (33)	5 (14)	<0.001
% of time in MVPA (≥ 2751)	2 (4)	0.7 (2)	<0.001

*Note*: Hip accelerometers were removed during the night; presence of weight loss includes weight loss in the previous 6 to 12 months; slow gait speed corresponds to <0.8 m/s.

Abbreviations: HABC, Health, Aging, and Body Composition Study; h, hour; IQR, interquartile range; ISS, I'm Still Standing Study; LASA, Longitudinal Ageing Study Amsterdam; min, minute; MVPA, moderate to vigorous physical activity; SB, sedentary behavior.

^†^ Chi‐square, t‐test or Mann–Whitney U test.

**Table 2:** Participants' characteristics according to appetite strata for cohorts with wrist‐worn accelerometers (*n* = 125)

Cohort: ISS	Good appetite (*n* = 90)	Moderate to poor appetite (*n* = 35)	
	Median (IQR)/%	Median (IQR)/%	*P*‐value^†^
Age (years)	86 (4)	86 (4)	0.605
Women	62%	57%	0.601
Weight loss	13%	31%	0.019
Living alone	69%	74%	0.529
Chronic conditions			0.519
None	13%	6%	
One	39%	44%	
Two or more	48%	50%	
Slow gait speed	40%	54%	0.149
Accelerometer wear time (min/day)	14 h 42 min (42 min)	14 h 36 min (43 min)	0.213
Average total daily counts (counts per minute)	1834 (728)	1889 (891)	0.916
Total activity			0.762
High tertile	68%	32%	
Middle tertile	76%	24%	
Low tertile	72%	28%	
Time in SB (0–2302) (h, min)	9 h 50 min (1 h 55 min)	9 h 30 min (2 h 54 min)	0.432
% of time in SB (0–2302)	66 (13)	64 (19)	0.612
Time in intensity 2303–4999 (h, min)	3 h 17 min (1 h 1 min)	3 h 28 min (1 h 30 min)	0.146
% of time in intensity 2303–4999	23 (7)	24 (12)	0.098
Time in intensity ≥5000 (min)	1 h 36 min (1 h 21 min)	1 h 21 min (1 h 29 min)	0.429
% of time in intensity ≥5000	11 (8)	9 (11)	0.493

*Note*: Physical activity and sedentary behavior calculated from 7:45 am to11:05 pm (15 h 20 min)/day; presence of weight loss includes weight loss in the previous 3 months; slow gait speed corresponds to <0.8 m/s.

Abbreviations: h, hour; IQR, interquartile range; ISS, I'm Still Standing Study; min, minute; SB, sedentary behavior.

^†^ Chi‐square, t‐test or Mann–Whitney U test.

Table 3 shows the association between device‐measured physical activity, sedentary behavior and appetite for studies with hip‐worn and wrist‐worn accelerometers. Among those with hip‐worn accelerometers, those with average counts within the highest tertile had greater than three times the odds of having good appetite after adjusting for age and sex (OR 3.04 (1.73–5.34)) and more than two times the odds in the fully adjusted model (OR 2.16 (1.15–4.06)) compared with those within the lowest tertile. The observed association between higher average counts and good appetite was even stronger among participants over 80 years old in models I (OR 3.43 (1.52–7.71)) and II (OR 3.24 (1.29–8.15)) but not in the fully adjusted model, where the association between good appetite and physical activity became non‐significant (OR 2.69 (0.90–8.02)), indicating that medical conditions and slow gait speed were more strongly correlated with physical activity than was appetite in the oldest old. For each percent increase in time spent per day in sedentary behavior, the odds for having good appetite decreased by 3% in all three models (OR 0.97 (0.95–0.996)), and with each percent increase of the day spent in light‐intensity physical activity, the odds for having good appetite increased by 2% (OR 1.02 (1.01–1.06)). Among participants using wrist‐worn accelerometers, no statistically significant association between device‐measured physical activity, sedentary behavior and appetite was observed.

**Table 3:** Association between good appetite and device‐measured movement characteristics

	Movement characteristic	Model I^¶^ OR (95% CI)	Model II^††^ OR (95% CI)	Model III^‡‡^ OR (95% CI)
Hip‐worn accelerometers (LASA and HABC)^§§^ (*n* = 1311)	Highest tertile of Avg counts/day^†^			
	All participants	3.04 (1.73–5.34)	2.91 (1.65–5.16)	2.16 (1.15–4.06)
	Participants ≥80 years old	3.43 (1.52–7.71)	3.24 (1.29–8.15)	2.69 (0.90–8.02)
	Cut‐points			
	% SB (0–173)^‡^	0.97 (0.95–0.98)	0.97 (0.95–0.995)	0.97 (0.95–0.996)
	% light intensity (174–2750)	1.05 (1.03–1.07)	1.04 (1.02–1.07)	1.02 (1.01–1.06)
	% MVPA (≥2751)	1.08 (0.99–1.19)	1.08 (0.99–1.19)	1.04 (0.95–1.15)
Wrist‐worn accelerometers (ISS) (*n* = 125)	Highest tertile of Avg counts/day	0.74 (0.26–2.11)	0.63 (0.21–1.92)	0.47 (0.13–1.64)
	Cut‐points			
	% SB (0–2302)^§^	1.02 (0.97–1.06)	1.02 (0.98–1.06)	1.02 (0.98–1.07)
	% intensity 2303–4999	0.94 (0.88–1.01)	0.95 (0.88–1.01)	0.95 (0.88–1.02)
	% intensity ≥ 5000	1.01 (0.95–1.08)	1.01 (0.94–1.07)	1.00 (0.93–1.07)

Abbreviations: HABC, Health, Aging, and Body Composition Study; ISS, I'm Still Standing Study; LASA, Longitudinal Ageing Study Amsterdam; MVPA, moderate to vigorous physical activity; OR, odds ratio; CI, confidence interval; SB, sedentary behaviour.

^†^ Tertiles of total activity were calculated for all participants and for participants over 80 years old, respectively.

^‡^ Additionally adjusted for % of time in MVPA for all three models.

^§^ Additionally adjusted for % of time in intensity ≥10 000 for all three models.

^¶^ Model I adjusted for age and sex.

^††^ Model II additionally adjusted for weight loss and living alone.

^‡‡^ Model III additionally adjusted for medical conditions and slow gait speed (<0.8 m/s).

^§§^ All models of hip‐worn accelerometers were adjusted for cohort (LASA or HABC).

## Discussion

Results from the present study show that participants with hip‐worn accelerometers, who were in the highest tertile for total activity and who engaged in more light‐intensity physical activity were more likely to have good appetite (2.16 (1.15–4.06) and 1.02 (1.01–1.06), respectively), while those who were more sedentary were less likely to have good appetite (0.97 (0.95–0.996)). In contrast, we observed no associations between appetite, physical activity and sedentary behavior among participants with wrist‐worn accelerometers.

Regulation of appetite is complex, and possible explanations of the association between appetite, physical activity and sedentary behavior could be linked to (i) the physical regulation of appetite relative to energy demands, and (ii) the effects of emotional well‐being on appetite sensation. Short‐term appetite control can be triggered by food intake (decreasing appetite to terminate energy intake), or fasting (stimulating appetite to initiate energy intake).^12^

Poor emotional well‐being and depressive symptoms have been identified as risk factors for poor appetite.^25^ Living alone, eating alone and being depressed may accelerate the progression of poor appetite.^12^ Physical activity has been shown to be effective in treating depression and improving well‐being.^26^ In addition, daily physical activity has been associated with higher quality of life in community‐dwelling adults,^27^ whereas time spent in sedentary behavior has been associated with a gradual decrease in health‐related quality of life.^27^ Thus, the association between appetite, physical activity and sedentary behavior may also be mediated by emotional well‐being.

While physical activity is an integral component of energy balance and is closely related to body composition, the effect of physical activity on appetite regulation and energy intake is debated. Previous findings have demonstrated that the age‐related reduction in the ability to acutely regulate energy intake is independent of habitual physical activity level.^28^ Nevertheless, when short‐term energy intake was evaluated (over 1 day), energy intake was more balanced in physically active than in sedentary older adults,^28^ an effect potentially mediated by more sensitive appetite regulation. Additionally, lower levels of physical activity have been identified as a risk factor for inadequate protein intake in older adults with poor appetite. Being more physically active and less sedentary has been reported to be associated with more fat‐free mass among older adults,^29^ while significantly higher fat mass was found in sedentary older adults.^30^ Hence, the differences in appetite between physically active and inactive older adults may partially be explained by the effects of activity level on body composition.

Studies among adult populations have identified that the relationship between physical activity intensity and energy expenditure is linear only at lower intensities and that total energy expenditure plateaus when physical activity is above the moderate activity level.^31^ If this holds for the older population, it could explain why accumulating light‐intensity physical activity increased the odds for having good appetite but why no association between moderate to vigorous physical activity (MVPA) and appetite was observed. Another possible explanation could be a lack of statistical power in the regression model (2% MVPA in the good appetite group and 0.7% MVPA in the moderate to poor appetite group).

Our results demonstrate a clear difference in the direction and magnitude of associations between physical activity, sedentary behavior and appetite by accelerometer placement, even after excluding participants below 80 years to match the age range between studies using hip‐ versus wrist‐worn accelerometers. Physical activity measured using hip‐worn accelerometers and that measured using wrist‐worn accelerometers was previously found to be at most moderately correlated among community‐dwelling older women. This may be explained by the lower sensitivity to distinguish activity intensity with wrist‐worn accelerometers.^32^ In addition, hip‐worn accelerometers generally outperform wrist‐worn accelerometers in predicting energy expenditure owing to their closeness to the center of gravity, which enables the movement of larger muscles to be captured.^33^ In contrast, a wide range of wrist movement can result in high accelerometer counts even when energy expenditure remains relatively low (e.g. when sitting).

A major strength of this study is the use of objective measures of movement patterns. The inclusion of multiple, established cohorts facilitated the investigation of different accelerometer placement locations and allowed for the adjustment for many covariates. The heterogeneity of our participants across three cohorts in terms of age and sex increases the generalizability of our results among community‐dwelling older people.

The study has some limitations. Appetite was harmonized across three cohorts from the original four‐ or five‐point scales into a dichotomous scale, which may reduce its variability. The purpose of using pragmatic cutpoints for wrist‐worn accelerometer data^19^ is to describe physical activity at relatively higher intensities when compared with sedentary behavior. However, these activity categories may not be directly comparable to moderate and vigorous intensity physical activity.

## Conclusion

Among community‐dwelling older adults, physical activity is associated with having good appetite, and sedentary behavior is associated with having moderate to poor appetite when activity is measured with hip‐worn accelerometers but not when it is measured with wrist‐worn accelerometers. This study highlights the potential of physical activity for the prevention of anorexia of aging as well as the importance of careful interpretation of accelerometer data from different body locations and concurrent health outcomes. Future longitudinal studies are warranted to clarify the temporal order of events between physical activity, sedentary behavior and changes in appetite among community‐dwelling older adults.

## Disclosure statement

All authors declare no conflicts of interest. This work was funded by the European Union Horizon 2020 PROMISS (Prevention Of Malnutrition In Senior Subjects) Project (grant agreement no. 678732). The Danish Diary Foundation supported S.F.B and the I'm Still Standing Study, and the Intramural Research Program at the National Institute on Aging, USA supported E.J.S.

## Supporting information


**Data S1.** Supporting Information.

## Data Availability

Data available on request due to privacy/ethical restrictions.
