# Supplementary material for: Associations between appetite, physical activity and sedentary behaviour from hip‐ and wrist‐worn accelerometers in community‐dwelling older adults
Source: Geriatr Gerontol Int. 2023 Apr 26;23(6):411–7. doi: 10.1111/ggi.14588 (PMC11503582; doi:10.1111/ggi.14588)
Supplement: Supplementary file 1 — Data S1. Supporting Information. [file GGI-23-411-s001.pdf]

Accelerometer data processing protocol

Participants from LASA and HABC wore the accelerometer on the right side of their waist with an elastic belt. Participants were instructed to remove the accelerometer during sleep, shower and other water-based activities. Participants from ISS were instructed to wear the accelerometer on their dominant wrist continuously, except when they took a shower or engaged in other water-based activities. Detailed instructions to record different behaviors in their accelerometer diary (e.g., sleep, wake and nap times) were provided and participants were encouraged to maintain their usual daily routines during the 7-day period.

Harmonization of accelerometer data across different brands and models used in the different cohorts was obtained by processing all raw acceleration data using the ActiLife software (version 6.4.11) into same ActiGraph counts metric (.gt3x file format) and converted into .agd file format without Low Frequency Extension Filter (1). Thereafter, the data files were analyzed using a 60-second epoch and produced counts per minute (cpm) in vector magnitude by a custom-made software (Propero) developed at the Department of Sports Science and Clinical Biomechanics, University of Southern Denmark. Valid data was defined as those with minimum 10-hour recordings for at least 4 days. Periods of >30 minutes (for wrist-worn accelerometers) or >60 minutes (for hip-worn accelerometers) of continuous zero counts allowing for one single spike below 100 counts was defined as non-wear time and set as missing in the analysis. To increase comparability between cohorts with different accelerometer placement and wear time set-up, data recorded between 11:05pm and 7:45am on wrist-worn accelerometers were removed. This reflects the average self-reported time in bed (2). Therefore, physical activity and sedentary behavior were summarized between 7:45 am to 11:05 pm (15 hr 20 min per day) for wrist-worn accelerometers and summarized between individual waking hours as recorded on the device for hip-worn accelerometers. Average daily counts were calculated and divided into tertiles. Physical activity and sedentary behavior were summed for the accelerometer wear time and time spent in the defined intensities of physical activity and sedentary behavior were calculated using accelerometer wear time as denominator and expressed in percentage.

In this study, physical activity intensity categories were defined according to cut-points in vector magnitude specifically for hip-worn and for wrist-worn accelerometers. Sedentary behaviour cut-points for both hip-worn (0-173 cpm) and wrist-worn accelerometers (0-2302 cpm) were reported in a calibration study using thigh-worn accelerometer (ActivPAL) as reference (3). The cut-point for moderate-to-vigorous activity (MVPA) for hip-worn accelerometers (≥ 2751 cpm) was validated with energy expenditure among older adults in the lab (4). Light intensity physical activity for hip-worn accelerometers was defined as intensity between above sedentary behavior and below MVPA (174-2750 cpm). There are currently no available cut-points above sedentary behaviour for wrist-worn accelerometers, and we have pragmatically decided to report activities at this intensity by ranges of 2303-5000, 5000-10000, and >10000 cpm (2).

Details regarding harmonization of appetite and covariates

Appetite

Appetite was harmonized across the three cohorts from the original four- or five-point scale into dichotomous "good appetite" and "moderate to poor appetite".

In LASA, appetite was assessed with the frequency for the following statement: "In the past week, I did not feel like eating, my appetite was poor" from the Dutch translation of the Centre for Epidemiologic Studies Depression scale (5). The responses could be: 1) rarely or never; 2) some of the time; 3) often; and 4) most of the time or always. Answers were dichotomized into moderate to poor (2-4) and good appetite (1).

The HABC assessed appetite four times throughout the year using question "In the past month, would you say that your appetite or desire to eat has been?". With response categories of very good, good, moderate, poor and very poor. Participants reporting very good or good appetite across all measurements were categorized as having good appetite. Participants reporting moderate or poor appetite at least once were categorized as having moderate to poor appetite.

ISS assessed appetite by the Short Nutritional Assessment Questionnaire (SNAQ) with the question "How would you describe your appetite" and answer options: very poor, poor, average, good, and very good (6). We transformed this variable into a dichotomous variable by collapsing options very poor, poor, and average into "moderate to poor appetite" and by collapsing options good and very good into "good appetite".

Other sociodemographic and health variables

Weight change during the past 6 months was self-reported in LASA, including amount of weight change in kilograms and then dichotomized into presence of weight loss during the past 6 months (yes or no). Weight change in HABC was calculated from self-reported weight between year 15 and year 16 of HABC measurement waves and then dichotomized into presence of weight loss during the past 12 months (yes or no). In ISS, weight loss during the past 3 months was self-reported by a question from the Mini Nutrition Assessment with four options: 1) weight loss greater than 3kg; 2) weight loss between 1 and 3kg; 3) no weight loss; and 4) does not know (7). This variable was dichotomized by collapsing options 1) and 2) into "yes" and options 3) and 4) into "no" for weight loss.

Living arrangement was self-reported in LASA and HABC by the question: "Besides yourself, how many other people live in your household or do you live alone?" and by the question:" Are you living alone?" in ISS. This variable was then dichotomized into living alone (yes or no). Age and sex were also collected in each study.

Physician-diagnosed medical conditions were self-reported from standardized interview questions and the number of conditions was calculated. Medical conditions included from LASA were the seven most common chronic diseases in the Netherlands: lung disease, cardiac disease, peripheral arterial disease, diabetes mellitus, stroke, osteoarthritis or rheumatoid arthritis and cancer (except non-melanoma skin cancer)(8). Seven medical conditions included from HABC were diagnosed by a physician in the past 6 months, including weak or failing kidneys, cardiovascular diseases, diabetes, stroke, cancer, pneumonia, and fractured bone(s)(9). Presence of medical conditions from a list of 17 items were asked in ISS. We combined some categories (e.g., blood clot, high blood pressure, heart failure, and myocardial infarction into cardiovascular diseases), then selected six medical conditions which closely resemble categories used from LASA and HABC and included them in this study: cardiovascular diseases, diabetes, renal diseases, respiratory diseases, arthritis, and osteoporosis. To harmonize medical condition across different studies, we transformed the continuous variable into a categorical variable with 1) none; 2) one; 3) two or more medical conditions.

Gait speed (m/s) was measured over 3 meters in LASA (maximal walking speed), over 20 meters in HABC (preferred walking speed) and as part of the short physical performance battery (SPPB) (10) over 3 meters in ISS (preferred walking speed). Presence of slow gait speed (yes or no) was defined as walking < 0.8 m/s (11)

Reference

1. Cain KL, Conway TL, Adams MA, Husak LE, Sallis JF. Comparison of older and newer generations of ActiGraph accelerometers with the normal filter and the low frequency extension. International Journal of Behavioral Nutrition and Physical Activity. 2013;**10**:51. https://doi.org/10.1186/1479-5868-10-51.
2. Tsai L-T, Boyle E, Brønd JC, Kock G, Skjødt M, Hvid LG*, et al.* Associations between objectively measured physical activity, sedentary behaviour and time in bed among 75+ community-dwelling Danish older adults. BMC geriatrics. 2021;**21**:1-8. https://doi.org/10.1186/s12877-020-01856-6.
3. Koster A, Shiroma EJ, Caserotti P, Matthews CE, Chen KY, Glynn NW*, et al.* Comparison of sedentary estimates between activPAL and hip-and wrist-worn ActiGraph. Medicine and science in sports and exercise. 2016;**48**:1514.
4. Santos-Lozano A, Santin-Medeiros F, Cardon G, Torres-Luque G, Bailon R, Bergmeir C*, et al.* Actigraph GT3X: validation and determination of physical activity intensity cut points. Int J Sports Med. 2013;**34**:975-982. 10.1055/s-0033-1337945.
5. Radloff LS. The CES-D scale: A self-report depression scale for research in the general population. Applied psychological measurement. 1977;**1**:385-401.
6. Wilson M-MG, Thomas DR, Rubenstein LZ, Chibnall JT, Anderson S, Baxi A*, et al.* Appetite assessment: simple appetite questionnaire predicts weight loss in community-dwelling adults and nursing home residents. The American journal of clinical nutrition. 2005;**82**:1074-1081.
7. Rubenstein LZ, Harker JO, Salvà A, Guigoz Y, Vellas B. Screening for undernutrition in geriatric practice: developing the short-form mini-nutritional assessment (MNA-SF). The Journals of Gerontology Series A: Biological Sciences and Medical Sciences. 2001;**56**:M366-M372.
8. Puts M, Lips P, Deeg D. Static and dynamic measures of frailty predicted decline in performance-based and self-reported physical functioning. Journal of clinical epidemiology. 2005;**58**:1188-1198. https://doi.org/10.1016/j.jclinepi.2005.03.008.
9. Brach JS, Simonsick EM, Kritchevsky S, Yaffe K, Newman AB. The association between physical function and lifestyle activity and exercise in the health, aging and body composition study. J Am Geriatr Soc. 2004;**52**:502-509. 10.1111/j.1532-5415.2004.52154.x.
10. Guralnik JM, Seeman T, Tinetti M, Nevitt M, Berkman L. Validation and use of performance measures of functioning in a non-disabled older population: MacArthur studies of successful aging. Aging Clinical and Experimental Research. 1994;**6**:410-419. https://doi.org/10.1007/BF03324272.
11. Cruz-Jentoft A, Baeyens J, Bauer J, Cruz-Jentoft A, Boirie Y, Cederholm T*, et al.* European Working Group on Sarcopenia in Older People. Sarcopenia: European consensus on definition and diagnosis: report of the European Working Group on Sarcopenia in Older People. Age. 2010. 10.1093/ageing/afq034.
